# Pharmacological Inhibition of Caspase-8 Suppresses Inflammation-Induced Angiogenesis in the Cornea

**DOI:** 10.3390/biom10020210

**Published:** 2020-01-31

**Authors:** Yunzhe Tian, He Li, Xiuxing Liu, Lihui Xie, Zhaohao Huang, Weihua Li, Zhuang Li, Yuan Pan, Xiaoqing Chen, Wenru Su

**Affiliations:** State Key Laboratory of Ophthalmology, Zhongshan Ophthalmic Center, Sun Yat-sen University, 528400 Guangzhou, China

**Keywords:** angiogenesis, corneal neovascularization, caspase-8, macrophage

## Abstract

Inflammation-induced angiogenesis is closely related to many diseases and has been regarded as a therapeutic target. Caspase-8 has attracted increasing attention for its immune properties and therapeutic potential in inflammatory disorders. The aim of our study is to investigate the clinical application of pharmacological inhibition of caspase-8 and the underlying molecular mechanisms in inflammation-induced angiogenesis in the cornea. A model of alkali burn (AB)-induced corneal neovascularization (CNV) in C57BL/6 wild-type (WT) mice and toll-like receptor 4 knockout (*Tlr4^-/-^*) mice was used. We found that AB increased caspase-8 activity and the pharmacological inhibition of caspase-8 exerted substantial inhibitory effects on CNV, with consistent decreases in caspase-8 activity, inflammatory cell infiltration, macrophage recruitment and activation, VEGF-A, TNF-α, IL-1β, MIP-1, and MCP-1 expression in the cornea. In vitro, caspase-8 mediated TLR4–dependent chemokines and VEGF-A production by macrophages. The TLR4 knockout significantly alleviated CNV, suppressed caspase-8 activity and downregulated expression of inflammatory cytokines and chemokines after AB. Taken together, these findings provide the first demonstration that the pharmacological inhibition of caspase-8 suppresses inflammation-induced angiogenesis and support the use of a pharmacological caspase-8 inhibitor as a novel clinical treatment for CNV and other angiogenic disorders.

## 1. Introduction

Angiogenesis is a fundamental process in growth. It is also vital for the pathogenesis of inflammation, cancer, atherosclerosis, rheumatoid arthritis, and ocular vascular diseases, such as diabetic retinopathy, age-related macular degeneration, and corneal neovascularization (CNV) [1,2,3,4,5,6,7]. Angiogenesis inhibition has been regarded as a therapeutic approach to treat these diseases [8,9,10,11,12,13]. Postnatal angiogenesis occurrence is usually associated with inflammation [1]. However, a deeper understanding of the molecular mechanisms that regulate inflammation-induced angiogenesis is needed, as are safe and effective methods of inhibiting angiogenesis.

Caspase-8, a member of caspase family with a well-characterized role in initiating cell apoptosis, has attracted increasing attention due to its immune properties [14,15,16]. It is a zymogen composed of an N-terminal prodomain and a C-terminal catalytic domain. Sequence Ile-Glu-Thr-Asp (IETD) can combine with caspase-8, which results in suppression of caspase-8 activity [17]. Several recent studies have demonstrated that caspase-8 plays an important role in the promotion of neuroinflammation, T-cell function, and cancer-related inflammation [18,19,20,21]. Furthermore, caspase-8 has recently been reported to mediate the activation of the Nod-like receptor family pyrin domain-containing 3 (NLRP3) inflammasome, comprising the NLRP3 scaffold, apoptosis-associated speck-like protein containing CARD (ASC) and caspase-1, which participates in various diseases, including autoimmune disorders, type 2 diabetes, atherosclerosis, gout, and obesity [15,22,23,24]. Our group’s most recent study found that caspase-8 is crucial in lymphangiogenesis and allograft rejection [25]. The pharmacological inhibition of caspase-8 repressed these pathological processes [25]. Moreover, Giulia et al. demonstrated that caspase-8 contributes to angiogenesis and growth in glioblastoma [26]. However, the involvement of caspase-8 in inflammation-associated angiogenesis and the underlying mechanisms are not yet clear.

Because the normal cornea lacks vessels and is convenient for experimental operation, it is an ideal system to investigate angiogenesis [27,28]. Furthermore, angiogenesis plays a key role in corneal disorders, such as corneal burn and virus infection [29,30,31]. Corneal alkali burn (AB) therefore serves as an ideal model to study inflammation-induced angiogenesis [32,33]. In our study, we used a mouse model of corneal AB to probe the application of a pharmacological caspase-8 inhibitor and the involved mechanisms in inflammatory CNV.

## 2. Materials and Methods

### 2.1. Animals

Total 180 adult male mice (six to eight weeks old), weighting 20–25 g were used in this study. 144 C57BL/6 wild-type (WT) mice were obtained from the Guangdong Medical Lab Animal Center. 36 C57BL/6-based homozygous toll-like receptor 4 knockout (*Tlr4^-/-^*, B6.B10ScNTlr4*^lps-del^*/JthJ) mice were purchased from the Model Animal Research Center of Nanjing University. The animals were housed under specific pathogen-free (SPF) conditions with a 12-h light/dark cycle and stable temperature (23 °C ± 2 °C) and humidity (55% ± 10%). All animal experiments were approved by the Institutional Animal Care and Use Committee of the Zhongshan Ophthalmic Center at Sun Yat-Sen University (No. 2017-094). The procedures were carried out according to the ARVO Statement for the Use of Animals in Ophthalmic and Vision Research.

### 2.2. Mouse Model of Corneal AB

The mouse model of corneal AB used here was performed by a slight modification previously described [34]. Briefly, a round piece of filter paper (2 mm in diameter) soaked with 5 μL of NaOH (1 M) was placed onto the right central cornea of each mouse for 30 s. Then, the cornea was rinsed extensively with 30 mL of sterile saline solution. Z-IETD-fmk is a cell-permeable, selective irreversible inhibitor of caspase-8 [17]. Z-IETD-fmk (20 μM, 40 μM, or 100 μM, Selleck, Houston, TX, USA) dissolved in dimethyl sulfoxide (DMSO) and diluted with saline solution was used [35]. The alkali-injured eye was topically treated with vehicle or the Z-IETD-fmk solution (5 μL) four times per day for 14 days.

### 2.3. Observation and Quantification of CNV

The eyes of the model mice (six mice/group) were examined by a slit-lamp microscopy (Zeiss, Jena, Germany), and photos were captured by a digital camera (Topcon, Tokyo, Japan) 3, 7, and 14 days after corneal AB. Macroscopic assessments were performed by two observers (technicians of animal experimental center) who have suitable experiences. They are not authors without prior knowledge of the allocation of the animals. According to previous studies, the percentage of CNV covered area (*A*%) was quantified by the following formula: *A*% = *C*/12 × 3.1416 [*r*^2^ − (*r* − *l*)^2^] × 100% (*C*: the clock hours of CNV coverage of the cornea, *l*: the average length of the vessels sprouted at each clock hour, and *r*: the radius of the mouse cornea, *r* = 1.5 mm) [36,37]. 

### 2.4. Histological Assessment of Mouse Eyes

Mice were euthanized on day 7 after corneal AB, and injured eyes were placed in 10% neutral buffered formalin at 25 °C for 24 h and then paraffin-embedded after dehydration with an ethanol gradient. Sagittal sections (4 µm) were stained with hematoxylin and eosin (H&E). For each sample, three planes were selected evenly within pupillary zone. Subsequently, three sections were selected from each plane. The inflammatory cells (identified by nuclear and cellular morphology from the local corneal cells) in the corneal stroma were counted manually at 20× magnification in five different fields of each H&E staining section. All the histological assessments were done as blind studies by the same two observers [24]. 

### 2.5. Immunofluorescence

Mice were sacrificed on day 7 after corneal AB for F4/80 immunostaining and on day 14 for CD31 immunostaining. The eyes were removed, fixed with 4% paraformaldehyde (PFA) overnight at 4 °C, and embedded in OCT. Sagittal sections (8 µm) were cut and blocked with 5% bovine serum albumin (BSA) in a Tris-buffered saline (TBS) buffer for 1 h at room temperature. Then, the sections were incubated with rat anti-F4/80 (Cat. No. Ab6640, 1:100, Abcam, Cambridge, UK) or rabbit anti-CD31 (Cat. No. Ab28364,1:50, Abcam) primary antibody overnight at 4 °C and further incubated with goat-anti-rat Alexa Fluor 555 (Cat. No. 4417, 1:1000, Cell Signaling Technology (CST), Danvers, MA, USA) or goat-anti-rabbit Alexa Fluor 555 (Cat. No. 4413, 1:1000, CST) secondary antibody for 1 h at room temperature. After counterstaining with DAPI (Abcam), the sections were examined with a fluorescence microscope (Nikon, Tokyo, Japan). We recognized the CD31^+^DAPI^+^ or F4/80^+^DAPI^+^ double immunostaining cells in the corneal stroma and count them at 20× magnification in five different fields of each immunostaining section manually. For each sample, three planes were selected evenly within the pupillary zone. Subsequently, three sections were selected from each plane. The counting work was all performed by the same two observers not aware of the experimental design and groups of study [25]. 

### 2.6. Cell Culture and Treatment

RAW264.7 (Zhong Qiao Xin Zhou Biotechnology, Shanghai, China), a murine macrophage cell line, was cultured in Dulbecco’s modified Eagle’s medium (DMEM) containing 10% fetal bovine serum (FBS) and 1% l-glutamine at 37 °C in a 5% CO_2_ atmosphere. Cells were pretreated with or without Z-IETD-fmk (10 μM) for 2 h and then stimulated with lipopolysaccharide (LPS, a TLR4 agonist, 100 ng/mL) for 8 h (for mRNA analysis) or 16 h (for caspase activity analysis) [25]. For cell viability, macrophages were treated with LPS and Z-IETD-fmk (1 μM, 5 μM, 10 μM, 20 μM, or 40 μM) for 24 h and assessed using a Trypan blue staining assay.

### 2.7. Real-Time Quantitative PCR

Total RNA was extracted from corneal tissue or cells lysates by using the RNA-Quick Purification Kit (Yishan Biotechnology, Shanghai, China), quantified with a NanoDrop spectrophotometer (Thermo, Waltham, MA, USA), and reverse transcribed into cDNA using HiScript II Q RT SuperMix for qPCR (Vazyme Biotechnology, Nanjing, China). The mRNA level was then determined by real-time PCR using ChamQ SYBR Color qPCR Master Mix (Vazyme Biotechnology). The relative changes in mRNA expression were determined by normalizing to the expression of GAPDH using the 2^−ΔΔCt^ method. The primer sequences were as follows: GAPDH forward, 5′-TGACCTCAACTACATGGTCTACA-3′ and reverse, 5′-CTTCCCATTCTCGGCCTTG-3′; vascular endothelial growth factor-A (VEGF-A) forward, 5′-GCACATAGAGAGAATGAGCTTCC-3′ and reverse, 5′-CTCCGCTCTGAACAAGGCT-3′; tumor necrosis factor alpha (TNF-α) forward, 5′-CAGGCGGTGCCTATGTCTC-3′ and reverse, 5′-CGATCACCCCGAAGTTCAGTAG-3′; interleukin-1 beta (IL-1β) forward, 5′-TTCAGGCAGGCAGTATCACTC-3′ and reverse, 5′-GAAGGTCCACGGGAAAGACAC-3′; NLRP3 forward, 5′-ATCAACAGGCGAGACCTCTG-3′ and reverse, 5′-GTCCTCCTGGCATACCATAGA-3′; ASC forward, 5′-GACAGTGCAACTGCGAGAAG-3′ and reverse, 5′-CGACTCCAGATAGTAGCTGACAA-3′; macrophage inflammatory protein-1 (MIP-1) forward, 5′-TTCTCTGTACCATGACACTCTGC-3′ and reverse, 5′-CGTGGAATCTTCCGGCTGTAG-3′; monocyte chemoattractant protein-1 (MCP-1) forward, 5′-TTAAAAACCTGGATCGGAACCAA-3′ and reverse, 5′-GCATTAGCTTCAGATTTACGGGT-3′; TLR4 forward, 5′-ATGGCATGGCTTACACCACC-3′ and reverse, 5′-GAGGCCAATTTTGTCTCCACA-3′.

### 2.8. Caspase-8 and Caspase-1 Activity

Total protein was obtained from corneal tissue or cell lysates by the Whole Cell Lysis Assay (KeyGen Biotech., Nanjing, China). Caspase-8 (Cat. No. K113) or caspase-1 (Cat. No. K111) activity of the corneas or cells was detected by the Colorimetric Assay Kit (BioVision, Milpitas, CA, USA) according to the manufacturer’s protocol. Briefly, 200 μg protein diluted to 50 μL cell lysis buffer was mixed with 50 μL 2X reaction buffer. Mixtures were incubated with IETD-p-nitroanilide (pNA) substrate (caspase-8) or YVAD-pNA substrate (caspase-1) in 96-plate well. After two hours of incubation at 37 °C, the pNA light emission is quantified using a spectrophotometer at 405 nm [25].

### 2.9. Statistical Analysis

One-way ANOVA with Tukey–Kramer post-hoc test was used to compare differences among three or more groups. Data analyses were performed using SPSS (22.0, IBM, Armonk, NY, USA). A value of *p* < 0.05 was accepted as statistically significant.

## 3. Results

### 3.1. Corneal AB Induced CNV and Increased Caspase-8 Activity

**Figure 1:**
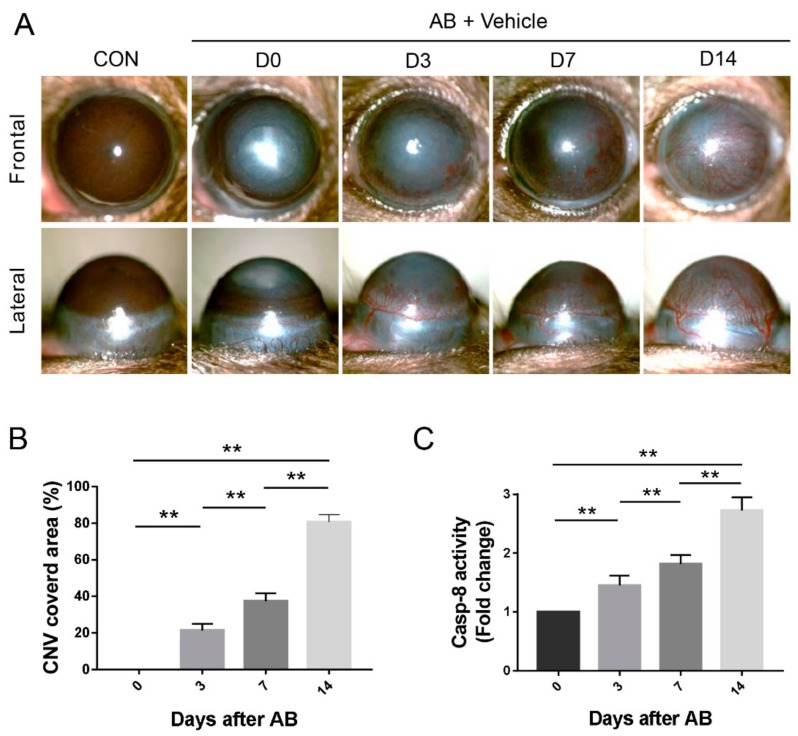
Corneal alkali burn (AB)-induced corneal neovascularization (CNV) and increased caspase-8 activity. (**A**) Representative photos of mouse eyes at 0, 3, 7, and 14 days after corneal AB injury. (**B**) Statistical analysis of the CNV covered area at various time-points (*n* = 6). (C) Colorimetric assay of caspase-8 activity in corneas (*n* = 3). The data are presented as the mean ± SD. ** *p* < 0.01. Abbreviations: CON, control; Casp, caspase.

In the AB model, exposure to NaOH (1 M) on the cornea resulted in time dependently increase in CNV. We found that limbal vessels sprouted, gradually grew into the cornea and reached the center on day 14 after corneal AB (*p* < 0.01, Figure 1A,B). The data also revealed that caspase-8 activity in the injured corneas was notably elevated and remained increased after corneal AB (*p* < 0.01, Figure 1C).

### 3.2. Pharmacological Inhibition of Caspase-8 Suppressed CNV after Corneal AB

**Figure 2:**
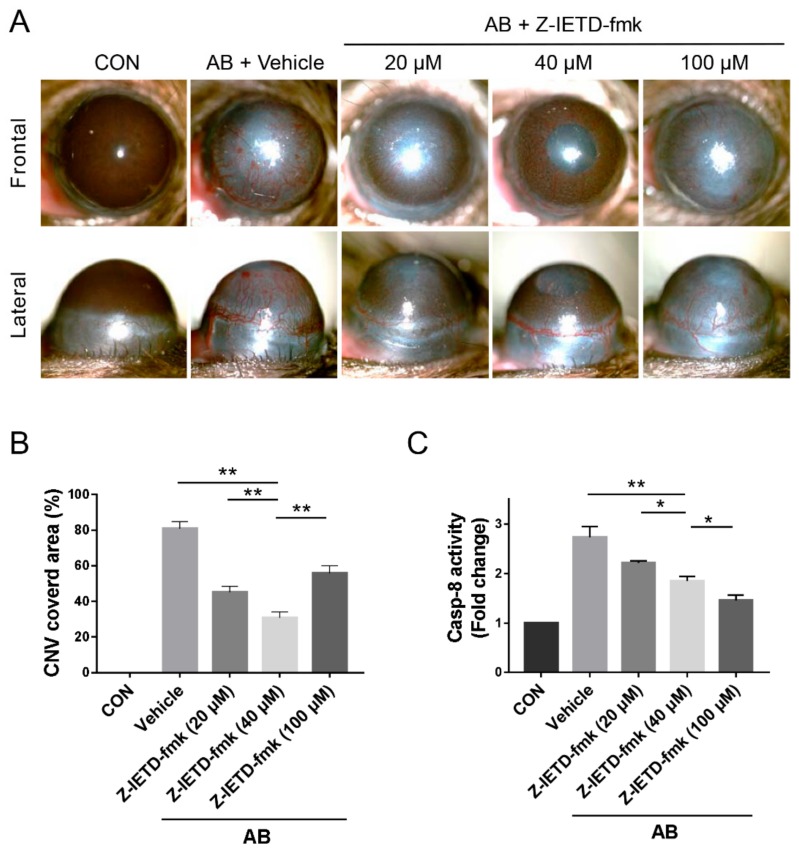
Pharmacological inhibition of caspase-8 suppressed CNV after corneal AB. (**A**) Representative images of the macroscopic CNV appearance after vehicle/Z-IETD-fmk treatment on day 14 after corneal AB injury. (**B**) Statistical analysis of the CNV covered area after vehicle/Z-IETD-fmk treatment (*n* = 6). (C) Colorimetric assay of caspase-8 activity in corneas (*n* = 3). The data are presented as the mean ± SD. * *p* <0.05, ** *p* < 0.01.

Then, we detected the potential role of a caspase-8 inhibitor in the development of CNV. When Z-IETD-fmk, a specific caspase-8 inhibitor, was topically applied for 14 days after alkali-induced injury, macroscopic CNV was noticeably reduced. We used dose curve to select the most effective dose of inhibitor and a maximum inhibitory effect on CNV was observed with the 40 μM dose (*p* < 0.01, Figure 2A,B). A dose of 40 μM was therefore used for subsequent experiments. Local administration of Z-IETD-fmk also resulted in the suppression of caspase-8 activity (20 μM vs. 40 μM, 40 μM vs. 100 μM, *p* < 0.05; vehicle vs. 40 μM, *p* < 0.01, Figure 2C).

### 3.3. Pharmacological Inhibition of Caspase-8 Suppressed CD31 and VEGF-A Expression after Corneal AB

**Figure 3:**
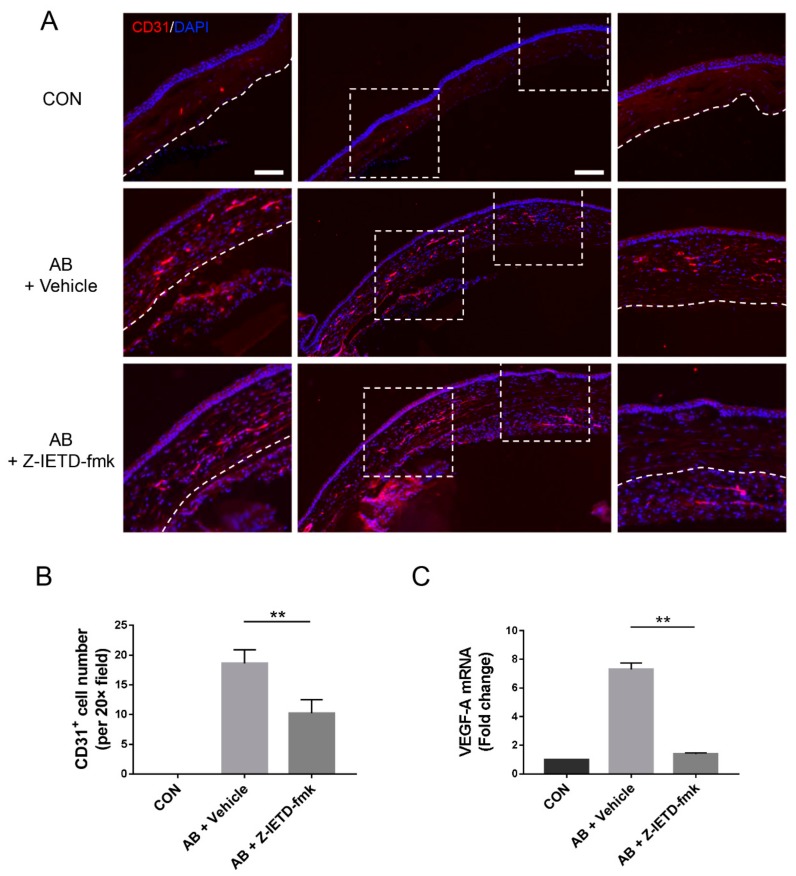
Pharmacological inhibition of caspase-8 suppressed CD31 and VEGF-A expression after corneal AB. (**A**) Immunostaining of frozen corneal sections for CD31. White boxed areas in the middle micrographs (10× field) are magnified in the left and right panels (20× field). Scale bars: 200 μm (middle), 100 μm (left and right). White dotted line: outline of the corneal epithelium. (**B**) Statistical analysis of the CD31-positive cell numbers (*n* = 5). (C) RT-qPCR analysis of VEGF-A mRNA expression in corneas (*n* = 3). The data are presented as the mean ± SD. ** *p* < 0.01.

Immunofluorescence analysis with an anti-CD31 antibody demonstrated a dramatic vascular area reduction in the experimental group compared with the vehicle group (*p* < 0.01, Figure 3A,B). Consistently, the mRNA expression of the proangiogenic factor VEGF-A was notably upregulated after corneal AB, whereas caspase-8 inhibition significantly reduced VEGF-A mRNA expression in injured corneas (*p* < 0.01, Figure 3C). These findings collectively suggest that caspase-8 plays a pivotal role in CNV and that caspase-8 inhibition is responsible for CNV suppression.

### 3.4. Pharmacological Inhibition of Caspase-8 Suppressed Alkali-Induced Inflammation

**Figure 4:**
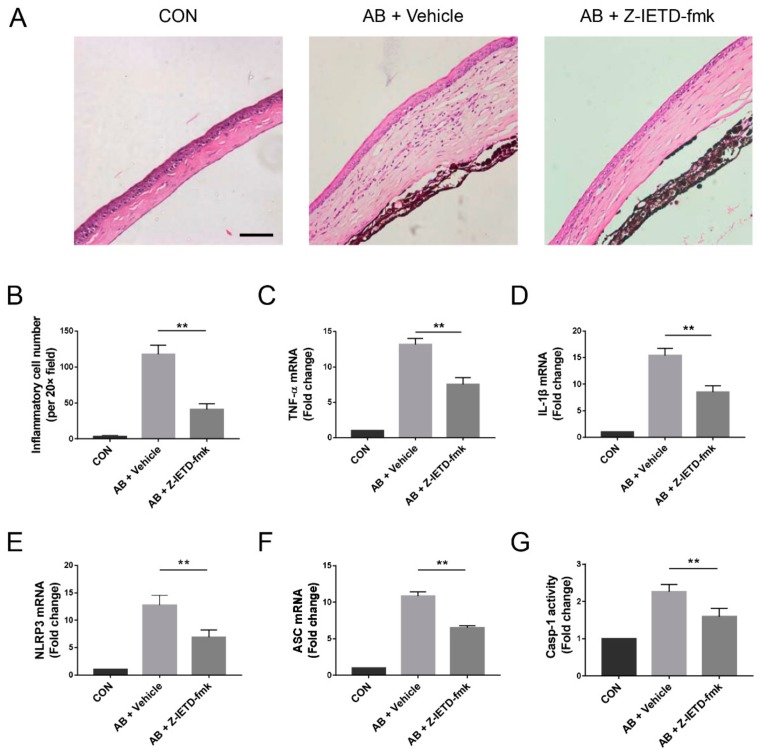
Pharmacological inhibition of caspase-8 suppressed alkali-induced inflammation and NLRP3 inflammasome activation. (**A**) Representative H&E staining of the eye tissue from different groups (20× field). Scale bars: 100 μm. (**B**) Histological analysis of inflammatory cell infiltration per 20× field (n = 5). (**C**–**F**) RT-qPCR analysis of TNF-α, IL-1β, NLRP3, and ASC mRNA expression in corneas (*n* = 3). (**G**) Colorimetric assay of caspase-1 activity in corneas (*n* = 3). The data are presented as the mean ± SD. ** *p* < 0.01.

To investigate the underlying mechanism of caspase-8 inhibition, we further analyzed the cornea sections. H&E staining clearly showed that inflammatory cell infiltration was markedly increased in the alkali-injured corneas. Topical application of Z-IETD-fmk reduced the number of inflammatory cells (*p* < 0.01, Figure 4A,B). The histological analysis of the corneal tissue supported the conclusion that the caspase-8 inhibitor suppressed inflammatory CNV. Correlatively, the RT-qPCR results revealed that the TNF-α and IL-1β mRNA levels were upregulated. However, the caspase-8 inhibitor reversed the upregulation of the mRNA expression of these inflammatory cytokines (*p* < 0.01, Figure 4C,D). We also found that NLRP3 and ASC mRNA expression and caspase-1 activity were increased. Local administration of the caspase-8 inhibitor markedly reduced these elevations (*p* < 0.01, Figure 4E–G).

### 3.5. Pharmacological Inhibition of Caspase-8 Suppressed Macrophage Recruitment

**Figure 5:**
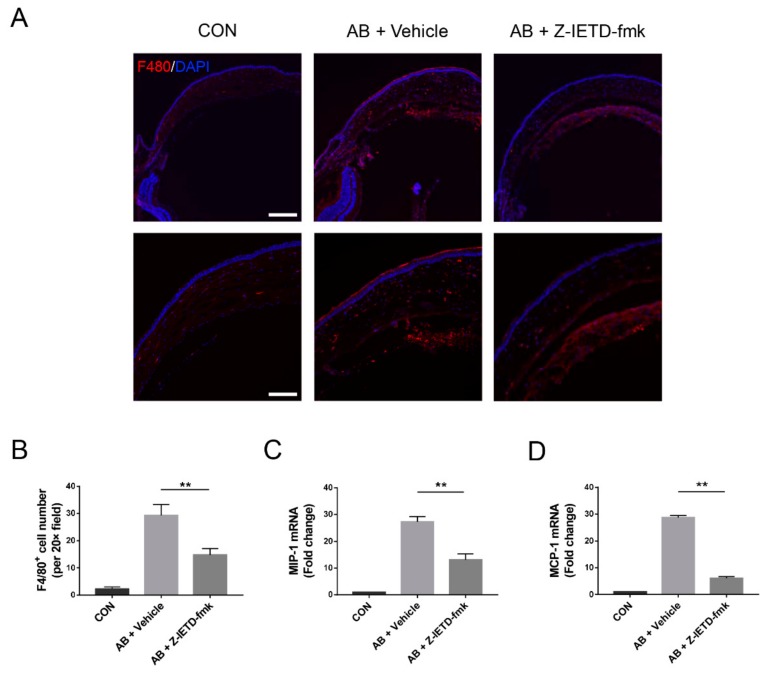
Pharmacological inhibition of caspase-8 suppressed macrophage recruitment. (**A**) Representative immunofluorescence micrographs of F4/80 staining. Scale bars: 200 μm (upper, 10× field), 100 μm (lower, 20× field). (**B**) Statistical analysis of F4/80-positive cell number (*n* = 5). (**C**,**D**) RT-qPCR analysis of MIP-1 and MCP-1 mRNA expression in corneas (*n* = 3). The data are presented as the mean ± SD. ** *p* < 0.01.

Macrophages are critically involved in CNV development [38,39,40,41]. Therefore, we subsequently investigated the role of caspase-8 in macrophage recruitment during CNV and examined the effects of caspase-8 inhibition on macrophage accumulation in alkali-injured corneas. F4/80^+^ macrophage numbers were notably increased in the injured corneas on day 7 after corneal AB and were significantly decreased with caspase-8 inhibition (*p* < 0.01, Figure 5A,B). The expressions of macrophage-related chemokines MIP-1 and MCP-1 were upregulated in the alkali-injured corneas and were noticeably reduced in the caspase-8 inhibitor-treated corneas (*p* < 0.01, Figure 5C,D), which indicated that caspase-8 inhibitor could suppress macrophage recruitment. The above observations suggest that caspase-8 signaling is important for F4/80^+^ macrophage recruitment and that this effect might be attributed to the caspase-8-mediated production of chemokines.

### 3.6. Pharmacological Inhibition of Caspase-8 Suppressed the Inflammatory Profiles of Macrophage In Vitro

**Figure 6:**
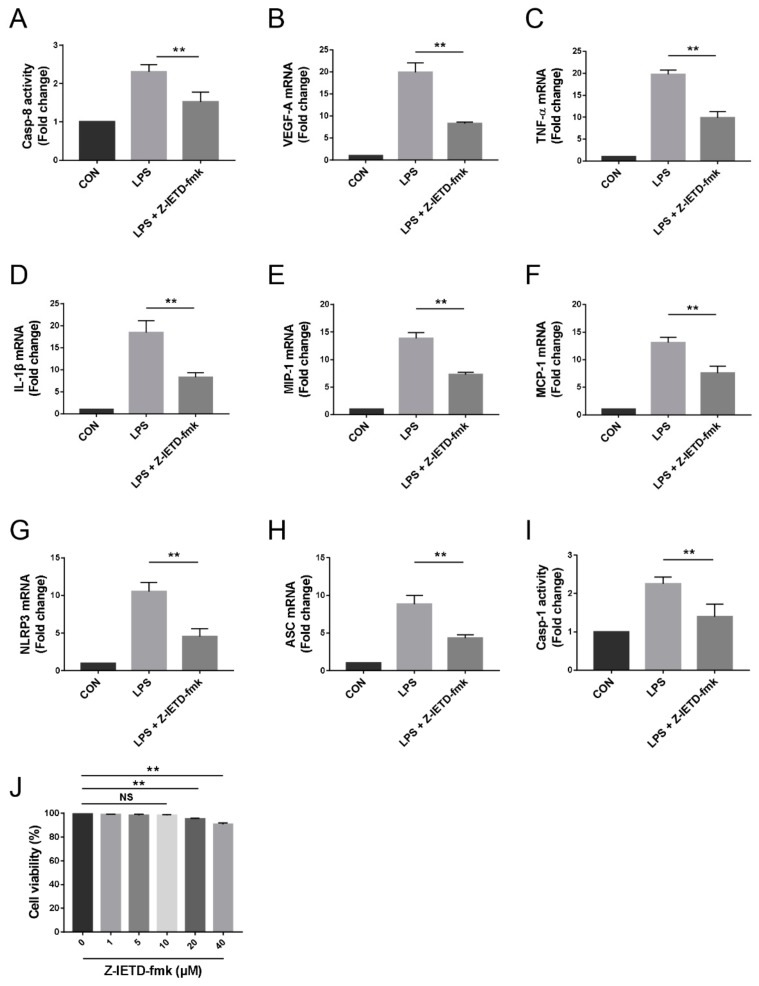
Pharmacological inhibition of caspase-8 suppressed the inflammatory profiles of RAW264.7 cells. (**A**) Colorimetric assay of caspase-8 activity in RAW264.7 cells (*n* = 3). (**B**–**H**) RT-qPCR analysis of VEGF-A, TNF-α, IL-1β, MIP-1, MCP-1, NLRP3, and ASC mRNA expression in RAW264.7 cells (*n* = 3). (I) Colorimetric assay of caspase-1 activity in RAW264.7 cells (*n* = 3). (**J**) Trypan blue exclusion assay for cell viability (*n* = 3). The data are presented as the mean ± SD. NS *p* > 0.05, ** *p* < 0.01.

We performed in vitro studies in the RAW264.7 murine macrophage cell line to further elucidate the roles of caspase-8, macrophages, and the NLRP3 inflammasome in CNV. Our results showed that LPS (100 ng/mL) significantly elevated caspase-8 activity and upregulated VEGF-A, TNF-α, IL-1β, MIP-1, MCP-1, NLRP3, and ASC mRNA expression as well as caspase-1 activity in macrophages. The caspase-8 inhibitor Z-IETD-fmk (10 μM), which did not affect cell viability (*p* > 0.05, Figure 6J), blocked the impact of LPS (*p* < 0.01, Figure 6A–I). Taken together, caspase-8 inhibition is able to block macrophage activation, contributing to the suppression of CNV.

### 3.7. Caspase-8 Activity was Partially Regulated by TLR4 Signaling

**Figure 7:**
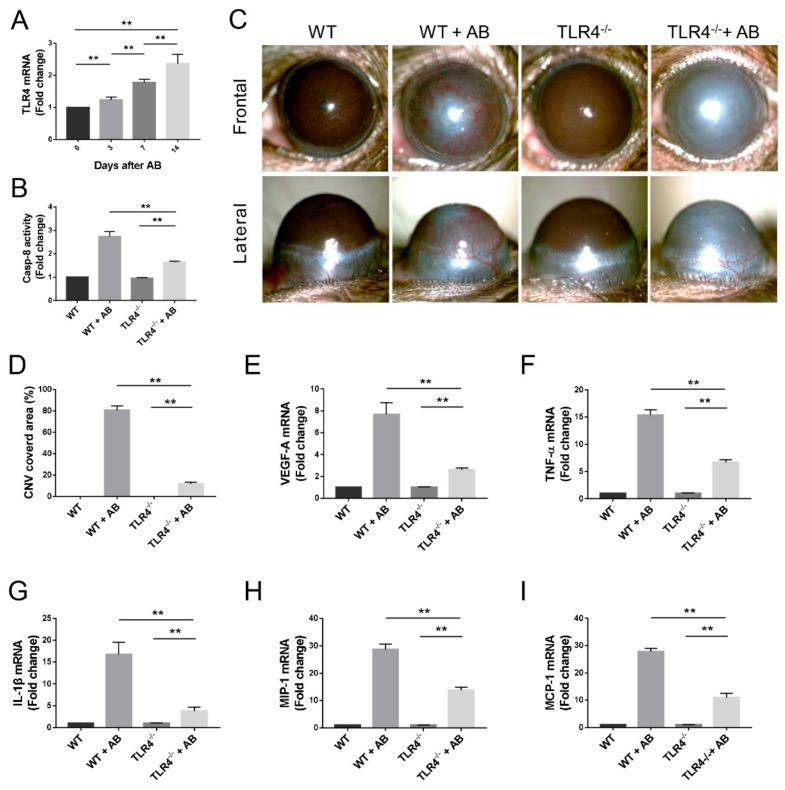
Caspase-8 activity is partially regulated by TLR4 signaling. (**A**) RT-qPCR analysis of TLR4 mRNA expression in corneas of WT mice at various time-points (*n* = 3). (**B**) Colorimetric assay of caspase-8 activity in corneas (*n* = 3). (**C**) Representative images of the macroscopic CNV appearance on WT and TLR4^-/-^ mice on day 14 after corneal AB injury. (**D**) Statistical analysis of the CNV covered area of WT and TLR4^-/-^ mice (*n* = 6). (**E**–**I**) RT-qPCR analysis of VEGF-A, TNF-α, IL-1β, MIP-1, and MCP-1 mRNA expression in corneas (*n* = 3). The data are presented as the mean ± SD. ** *p* < 0.01.

Our in vitro experiments showed that a TLR4 agonist stimulated caspase-8 activity, and caspase-8 inhibition blocked TLR4-depentent macrophage activation. We, thus, investigated whether TLR4 signaling regulates caspase-8 activity in CNV after AB. TLR4 mRNA expression was significantly increased in the alkali-burned corneas of WT mice and remained elevated 14 days after AB (*p* < 0.01, Figure 7A). Using *Tlr4^-/-^* mice, our results show that caspase-8 activity and CNV in the injured corneas was increased after AB. TLR4 knockout noticeably but only partially reduced caspase-8 activity after AB (*p* < 0.01, Figure 7B). Furthermore, CNV was significantly reduced in *Tlr4^-/-^* mice in comparison with WT mice (*p* < 0.01, Figure 7C,D). Consistently, the RT-qPCR results revealed that the VEGF-A, inflammatory cytokines, and chemokines mRNA levels were elevated after AB in *Tlr4^-/-^*mice but were downregulated comparing with the WT mice (*p* < 0.01, Figure 7E–I). Therefore, TLR4 signaling plays an important role in the activation of caspase-8 in CNV after AB.

## 4. Discussion

This study shows that pharmacological caspase-8 inhibition is a feasible and effective treatment strategy for CNV and identifies the key role of caspase-8 in CNV after corneal AB. We found that corneal AB induced CNV and increased caspase-8 activity and that caspase-8 inhibition had substantial suppressive effects on CNV, with consistent decreases in caspase-8 activity, inflammatory cell infiltration, F4/80^+^ macrophage recruitment and activation, VEGF-A, inflammatory cytokines and chemokines expression in the cornea after corneal AB. In vitro, a caspase-8 inhibitor reversed the LPS-induced increases in VEGF-A, TNF-α, IL-1β, MIP-1, and MCP-1 mRNA expression in macrophages. Taken together, our current findings demonstrate the therapeutic effects of the pharmacological caspase-8 inhibition on CNV and elucidate a novel mechanism whereby caspase-8 promotes CNV in corneal AB.

Caspase-8, which is well acknowledged as the initiator of apoptotic signaling, has also been shown to possess numerous immune properties [18,19,20,21]. Burguillos et al. found that caspase-8 mediates TLR4-dependent microglial activation and neuroinflammation [18]. Kim et al. and Qi et al. reported that caspase-8 promotes inflammation in cancer and asthma, respectively [20,21]. Recently, our group showed that caspase-8 promotes inflammatory lymphangiogenesis and allograft rejection in corneal transplantation [25]. Moreover, it has been demonstrated that the caspase-8 promotes NF-κB pathway activation to control microglia activation and IL-1β, TNF-α production to promote neuroinflammation [18]. A study found that caspase-8 activates NLRP3 inflammasome and increases IL-1β production in acute glaucoma [24]. Therefore, caspase-8 may upregulate inflammatory cytokines expression to promote inflammation via the NF-κB pathway or/and NLRP3 inflammasome. In the present study, we made new observations, namely, that caspase-8 activity is increased in the cornea after corneal AB and that caspase-8 inhibition suppresses CNV. In line with previous studies, we found that caspase-8 activity is increased concurrently with increased NLRP3 inflammasome activity and relevant cytokines/chemokines expression, whereas the blockade of caspase-8 prevents NLRP3 inflammasome activation and decreases those cytokines/chemokines expression in the cornea after corneal AB. LPS also significantly elevates caspase-8 activity, NLRP3 inflammasome activity, and relevant cytokines/chemokines expression in vitro. These findings suggest that caspase-8 is a crucial mediator of CNV in corneal AB, and caspase-8 may affect those cytokines/chemokines expression through the NF-κB pathway or/and NLRP3 inflammasome.

Macrophages play critical roles in angiogenesis [38,39,40,41]. During angiogenesis, macrophages differentiate into an angiogenic phenotype. Activated macrophages secrete proangiogenic factors, inflammatory cytokines, and chemokines, which provoke the division and proliferation of preexisting vascular endothelial cells and recruit more macrophages to the inflamed tissue or tumor [38,39,40,41]. The present study showed that by blocking caspase-8 with an inhibitor, it might be possible to reduce the recruitment of macrophages and decrease the expression of MIP-1 and MCP-1, which have been shown to play critical roles in the recruitment of these cells [42]. Notably, the subsequent decrease in macrophage recruitment is consistent with the inhibitory effects on VEGF-A, TNF-α, and IL-1β expression. Furthermore, in vitro, caspase-8 inhibition also leads to decreased VEGF-A, TNF-α, IL-1β, MIP-1, and MCP-1 expression in RAW264.7 cells stimulated with LPS. These results suggest that corneal AB may first induce resident corneal cell injury and/or activation to release MCP-1 and MIP-1, which then recruit and activate macrophages. Eventually, activated macrophages may produce VEGF-A, TNF-α, IL-1β, MIP-1, MCP-1, and other mediators to further promote CNV.

Toll-like receptor 4 (TLR4) is a key pattern recognition receptor (PRR) that recognizes molecular patterns associated with microbial pathogens and damage-associated molecular pattern (DAMP) molecules [43,44]. HMGB1, an endogenous ligand of TLR4, can drive the pathogenesis of inflammatory and angiogenic diseases and immune regulation, such as CNV, ischemia-reperfusion injury and hemorrhagic shock, though HMGB1/TLR4 signaling pathway [45,46,47,48]. TLR4/caspase-8 signaling pathway also plays pivotal roles in various inflammation [24,49,50]. Chi et al. reported that TLR4/caspase-8 pathway activation results in the maturation of IL-1β and aggravates the inflammation in acute glaucoma [24]. Shenderov et al. showed that, in the situation of endoplasmic reticulum stress, TLR4 activation stimulates IL-1β expression regulated by caspase-8 [49]. Philip et al. revealed that caspase-8 mediates the induction of inflammatory cytokines caused by a bacterial infection and TLR activation [50]. Recently, it has been reported that HMGB1/TLR4 mediates caspase-8 activation to promote allograft rejection in corneal transplantation [25]. In our study, we found that the absence of TLR4 is associated with downregulated caspase-8 activation, indicating that TLR4 is one of the upstream signals of caspase-8. Taken together, it is possible that the corneal AB initially leads to corneal cell damage. DAMP molecules released from damaged cells, such as HMGB1, combines with TLR4 to activate caspase-8 signaling promoting CNV in the cornea. However, we found that knockdown of TLR4 can reduce caspase-8 activity, but not abrogate the increase of caspase-8. It has been reported that besides TLR4, HMGB1 can activate other TLRs like TLR2 and receptors for advanced glycation end products (RAGE), leading to the production and secretion of pro-inflammatory cytokines [51,52]. On the other hand, Chi et al. demonstrated that HMGB1/capase-8 pathway promotes the activation of NF-κB, which subsequently induces the processing of IL-1β and aggravates the inflammation [53]. Therefore, it is possible that caspase-8 is also regulated by other signals, such as TLR2, during the inflammation in AB model, and TLR4 is only partially responsible for caspase-8 activity.

Recently, Ueta et al. demonstrated that in pathological angiogenesis mouse model, RIPK1 abundantly expresses in infiltrating macrophages and inhibition of RIPK1 alleviates angiogenesis. Furthermore, caspase inhibition can augment RIPK1 activation, which cause aggravation of pathological angiogenesis [54]. Similarly, during the study, we found that 100 μM Z-IETD-fmk treatment decreases the activity of caspase-8 but aggravates CNV comparing with the 40 μM group. Therefore, it is possible that high concentration of Z-IETD-fmk suppresses caspase-8 activity, resulting in inhibition of RIP kinase activation and aggravation of pathological angiogenesis. In this study, we focus on the function of caspase-8 during inflammation-induced angiogenesis. However, the mechanism of caspase-8-mediated inflammation is largely unclear and needed to be explored in further study.

In conclusion, this study, to the best of our knowledge, presents a new mechanism by which caspase-8 promotes inflammation-induced angiogenesis and reveals for the first time that the pharmacological inhibition of caspase-8 suppresses inflammation-induced angiogenesis. They support the use of a pharmacological caspase-8 inhibitor as a novel clinical treatment for CNV and other angiogenic disorders.

## Abbreviations

AB	alkali burn
CNV	corneal neovascularization
NLRP3	Nod-like receptor family pyrin domain-containing 3
ASC	apoptosis-associated speck-like protein containing CARD
WT	wild-type
TLR4	toll like receptor 4
SPF	specific pathogen-free
PFA	paraformaldehyde
BSA	bovine serum albumin
TBS	Tris-buffered saline
DMEM	Dulbecco’s modified Eagle’s medium
FBS	fetal bovine serum
LPS	lipopolysaccharide
VEGF-A	vascular endothelial growth factor-A
TNF-α	tumor necrosis factor alpha
IL-1β	interleukin-1 beta
MIP-1	macrophage inflammatory protein-1
MCP-1	monocyte chemoattractant protein-1
CON	control
Casp	caspase
